# Synthesis of potential metal-binding group compounds to examine the zinc dependency of the GPI de-*N*-acetylase metalloenzyme in *Trypanosoma brucei*

**DOI:** 10.1016/j.carres.2011.02.004

**Published:** 2011-05-01

**Authors:** Nuha Z. Abdelwahab, Michael D. Urbaniak, Michael A.J. Ferguson, Arthur T. Crossman

**Affiliations:** Division of Biological Chemistry and Drug Discovery, College of Life Sciences, The University of Dundee, DD1 5EH Dundee, Scotland, United Kingdom

**Keywords:** Glycosylphosphatidylinositol (GPI) biosynthesis, Zinc metalloenzyme inhibitor, Zinc-binding group, Branched monosaccharides, Phosphatidylinositol de-*N*-acetylase

## Abstract

A small zinc-binding group (ZBG) library of deoxy-2-C-branched-monosaccharides, for example, 1,5-anhydroglucitols, consisting of either monodentate ligand binding carboxylic acids or bidentate ligand binding hydroxamic acids, were prepared to assess the zinc affinity of the putative metalloenzyme 2-acetamido-2-deoxy-α-d-glucopyranosyl-(1→6)-phosphatidylinositol de-*N*-acetylase (EC 3.5.1.89) of glycosylphosphatidylinositol biosynthesis. The *N*-ureido thioglucoside was also synthesised and added to the ZBG library because a previous *N*-ureido analogue, synthesised by us, had inhibitory activity against the aforementioned de-*N*-acetylase, presumably via the *N*-ureido motif.

## Graphical abstract

**Figure:**
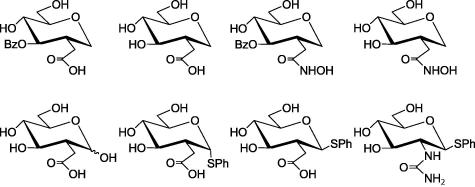

## 1 Introduction

Glycosylphosphatidylinositol (GPI) acts as a membrane anchor for a small but significant proportion of higher eukaryote cell-surface glycoproteins that are particularly abundant in protozoan parasites such as *Trypanosoma brucei*, the causative agent of African sleeping sickness in humans and the related disease Nagana in cattle.^1^ The structure, biosynthesis, and function of GPI anchors and related molecules have been extensively reviewed.^1^, ^2^, ^3^, ^4^ Disruption of GPI biosynthesis in the clinically relevant bloodstream form of *T. brucei* has been genetically^5^, ^6^, ^7^, ^8^ and chemically^9^ validated as a drug target.

**Figure 1:**
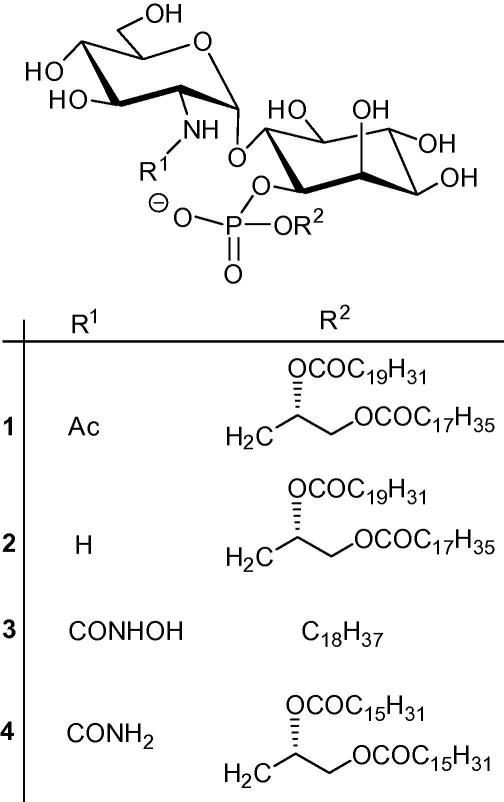

A key early step in the biosynthesis of the GPI anchors is the de-*N*-acetylation of 2-acetamido-2-deoxy-α-d-glucopyranosyl-(1→6)-phosphatidylinositol^10^ [α-d-Glc*p*NAc-PI (**1**, Fig. 1)] to form α-d-Glc*p*NH_2_-PI (**2**, Fig. 1). De-*N*-acetylation is a prerequisite for subsequent processing of **2** that leads to mature GPI anchor precursors.^11^ In *T. brucei*, de-*N*-acetylation is followed by mannosylation and subsequent inositol-acylation of **2,** whereas in mammalian cells the order of these reactions is reversed.^12^, ^13^

**Figure 2:**
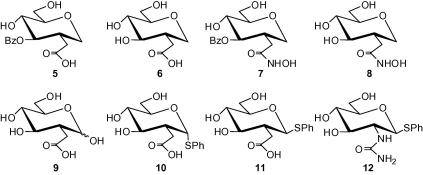
A small library of zinc chelator probes.

Previously, we have shown^14^ that mammalian and trypanosomal α-d-Glc*p*NAc-PI de-*N*-acetylases are zinc metalloenzymes, proposed a mechanism of action similar to that of zinc peptidases and postulated that known zinc binding motifs^15^, ^16^ such as the *N*-hydroxyurea analogue **3** (Fig. 1),^17^ could act as inhibitors. Here, we have designed and synthesised a small library of deoxymonosaccharides [**5**–**12** (Fig. 2)] containing recognisable zinc binding groups (ZBGs), that is, carboxylic acids and hydroxamic acids, as well as a potentially new ZBG, the ureido derivative, that should continue to probe the trypanosomal α-d-Glc*p*NAc-PI de-*N*-acetylase.

A good starting point for our compound library was the earlier work by Hindsgaul and co-workers^18^, ^19^ which demonstrated the effectiveness of 1,5-anhydro-2-deoxy-d-glucitol hydroxamic acids, for example **7**,^19^ as ZBG probes. The hydroxamic acid **7** was resynthesised and included in the compound library because **7** was shown to be a potent inhibitor of LpxC,^19^ presumably via zinc chelation, and could serve as the standard by which to compare the potency of the other analogues in the library. Therefore, compounds **5**, **6** and **8**† resemble those of Hindsgaul et al. whereby the 2-C appendage is either a hydroxamic acid or a carboxylic acid ZBG moiety. Compounds **9**–**11** were synthesised to supply potential glycosyl donors for another project but might also exhibit some degree of inhibition towards the trypanosome de-*N*-acetylase enzyme. Lastly, the *N*-ureido thioglycoside **12** was fashioned because of previous inhibitory data of the *N*-ureido-GlcNAc-PI derivative **4**^20^ (Fig. 1) against the trypanosome de-*N*-acetylase enzyme. Analogue **12** is a truncated version of **4** which focuses on, what we believe to be the most potent inhibitory component of **4**, the *N*-ureido motif.

## 2 Results and discussion

**Scheme 1:**
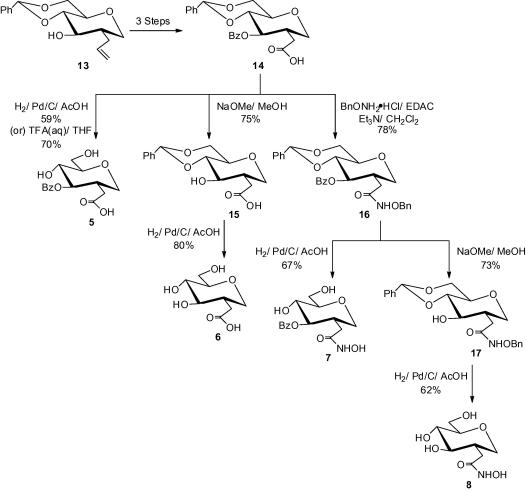

The synthesis, of the analogues **5**–**8**, is based on a successful approach^18^, ^19^ used previously (Scheme 1).

The first three steps, benzoylation→ozonolysis→Pinnick^21^ oxidation, from the known^18^ 2-*C*-allyl derivative **13** was accomplished straightforwardly to furnish the pivotal carboxylic acid **14**.^19^ The carboxylic acid analogue **14**^19^ and the corresponding intermediates from **13**^18^ were not fully characterised in the literature. Consequently, we have included the analytical data for those intermediates, and that of compound **14**,^19^ in this paper as Supplementary data. Hydrogenolysis of the benzylidene protecting group of compound **14** furnished the target analogue **5** in 59% yield; alternatively, the yield could be improved to 70% by using aqueous TFA.

The synthesis of carboxylic acid **6** emerged from the de-*O*-benzoylation of **14**,^19^ under Zemplén conditions, followed by hydrogenolysis over 10% palladium on carbon to give the crude derivative **6** (Scheme 1). The analogue **6** was then purified by reversed phase chromatography (RPC) to afford the final target glucitol **6** in 80% yield.

The carboxylic acid derivative **14**^19^ was coupled with *O*-benzylhydroxylamine hydrochloride (BnONH_2_·HCl) using *N*-(3-dimethylaminopropyl)-*N*′-ethylcarbodiimide hydrochloride (EDAC) to give the known^19^ hydroxyamide **16** (see the Supplementary data for the analytical data of **16**). The benzyloxyamide **16** was hydrogenated, as described in the literature, to give the hydroxamic acid **7**;^19^ ^1^H NMR assignments for **7** were identical to those reported in the literature^19^ and see the Supplementary data for the ^13^C NMR assignments of **7**. The ZBG analogue **8** was synthesised following the sequence **16**→**17**^18^→**8**, as previously described for **6**. An alternative synthesis of the derivative **17**^18^ is described in the Supplementary data.

**Scheme 2:**
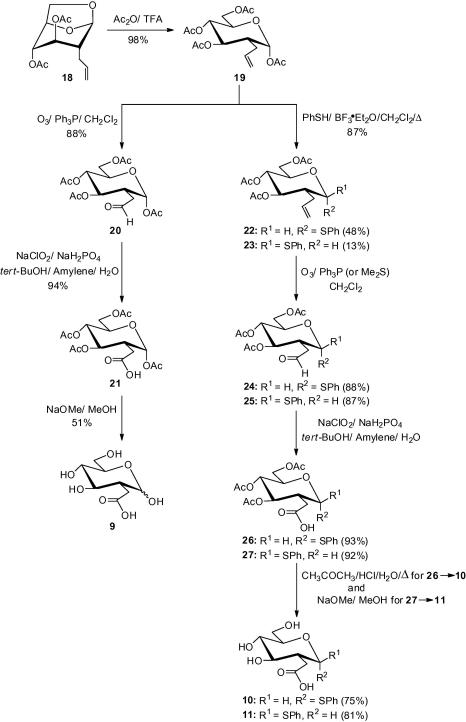

The synthesis of the targeted carboxylic acid **9** (Scheme 2) began from the acetolysis of the 1,6-anhydro derivative **18**^22^ to give, exclusively, the α-2-*C*-allyl derivative **19** [*J*_1,2_ = 3.1 Hz]. The tetraacetate derivative **19** proved to be a very useful intermediate because **19** could be altered to supply analogues **10** and **11**, as well. Thus, a portion of the 2-*C*-allyl intermediate **19** was ozonised to give the aldehyde **20**, which was oxidised, following Pinnicks’ protocols,^21^ to furnish the carboxylic acid **21** in 94% yield. Lastly, the tetraacetate **21** was de-*O*-acetylated with 0.03 M methanolic sodium methoxide to produce the fully deprotected carboxylic acid analogue **9** in 51% yield, as a mixture of α/β anomers.

Another portion of the 2-*C*-allyl derivative **19** was transformed into the corresponding α- and β-phenylthioglucosides **22** and **23**, respectively, via Lewis acid (BF_3_·Et_2_O) catalysed substitution of the anomeric acetate with thiophenol in refluxing dichloromethane.^23^ These two anomers were separated by radial band chromatography to furnish the α-anomer **22** (*J*_1,2_ = 4.9 Hz) and the β-anomer **23** (*J*_1,2_ = 10.9 Hz) in 48% and 13% yields, respectively. The closing sequences **22**→**24**→**26**→**10** and **23**→**25**→**27**→**11** were then conducted without incident, essentially as those described for **9**; the exception being **26**→**10** which was achieved via acid hydrolysis^24^ (Scheme 2).

**Scheme 3:**
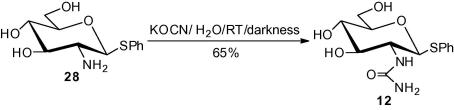

A synthesis of 1-thiophenyl-2-deoxy-2-ureido-β-d-glucopyranoside **12** was obtained on treatment of the known amine^25^ **28** with potassium cyanate (KOCN) and water at room temperature in total darkness^26^, ^27^ (Scheme 3). After evaporation to dryness, the crude ureido compound was purified by reversed phase chromatography to give crystalline **12** (65% yield; characteristic ^13^C carbonyl carbon at *δ* 158.47 ppm).

Details of the results of enzymatic studies with the above ZBG analogues will be reported elsewhere in due course.

## 3 Experimental

### 3.1 General methods

^1^H, ^13^C, ^31^P NMR spectra were recorded on a Bruker AVANCE 500 MHz spectrometer using deuteriochloroform as a solvent and tetramethylsilane as the internal standard, unless otherwise indicated. All coupling constants (*J*) are given in Hertz. High resolution electrospray ionisation mass spectra (HRESIMS) and liquid chromatography mass spectra (LCMS) were recorded with a Bruker microTof spectrometer. Melting points were determined on a Reichert hot-plate apparatus and are uncorrected. Optical rotations were measured with a Perkin–Elmer 343 polarimeter. Thin layer chromatography (TLC) was performed on Kieselgel 60 F_254_ (Merck) or RP-18 F_254s_ (Merck) plates with various solvent systems as developers, followed by detection under UV light or by charring using either sulfuric acid–water–ethanol (15:85:5), phosphomolybdic acid, orcinol or ninhydrin spray reagents. Flash column chromatography (FCC) was performed on Kieselgel 60 (0.040–0.063 mm) (Merck). Reversed phase chromatography was performed on a C18 cartridge supplied by Sigma–Aldrich. Radial-band chromatography (RBC) was performed using a Chromatotron (model 7924T, TC Research UK) with silica gel F_254_ TLC standard grade as the adsorbent. All reactions were carried out in commercially available dry solvents, unless otherwise stated. Light petroleum refers to the fraction having a boiling range 60–80 °C, unless indicated otherwise.

### 3.2 Synthesis of the ZBG library

#### 3.2.1 1,5-Anhydro-3-*O*-benzoyl-2-*C-*carboxymethyl-2-deoxy-d-glucitol (**5**)

##### 3.2.1.1 Method A

A solution of the benzylidene compound **14**^19^ (20 mg, 0.05 mmol) in AcOH (2 mL) containing 10% palladium on carbon (10 mg) was stirred under a slight overpressure of hydrogen at room temperature for 4 h. The reaction mixture was filtered through a pad of Celite and concentrated under reduced pressure. The residue was purified by FCC (10:1:0.02 CHCl_3_–MeOH–AcOH) to furnish a brown paste **5** (9 mg, 59%), which was indistinguishable from that obtained by the following procedure.

##### 3.2.1.2 Method B

A solution of the benzylidene compound **14**^19^ (40 mg, 0.10 mmol) in THF (2 mL) and 96% (aq) TFA (0.5 mL) was stirred at room temperature for 3 h. The reaction mixture was concentrated under reduced pressure and co-evaporated with toluene (2 × 5 mL). The residue was purified with the same solvent system as in method A to give the acid **5** (21 mg, 70%): *R*_f_ 0.20 (10:1:0.02 CHCl_3_–MeOH–AcOH); $[\alpha ]{\;}_{\text{D}}^{25}$ +8.9 (*c* 1.0, MeOH); ^1^H NMR (CD_3_OD**,** 500 MHz): *δ* 8.10–7.49 (m, 5H, Ph), 5.10 (dd, 1H, *J*_2,3_ 10.7, *J*_3,4_ 9.3 Hz, H-3), 4.10 (dd, 1H, *J*_1a,2_ 4.7, *J*_1a,1b_ 11.5 Hz, H-1a), 3.88 (dd, 1H, *J*_5,6a_ 2.1, *J*_6a,6b_ 11.8 Hz, H-6a), 3.71 (dd, 1H, H-6b), 3.60 (t, 1H, *J*_4,5_ 9.3 Hz, H-4), 3.40 (t, 1H, *J*_1a,1b_ 11.5 Hz, H-1b), 3.37–3.34 (m, 1H, H-5), 2.50–2.41 (m, 1H, H-2), 2.35 (dd, 1H, *J*_2,7a_ 4.8, *J*_7a,7b_ 16.0 Hz, H-7a), 2.17 (dd, 1H, H-7b); ^13^C NMR (CD_3_OD, 125 MHz): *δ* 175.49 (C=O), 168.15 (Ph*C*O), 134.29–129.56 (C-Ph), 82.75 (C-5), 79.92 (C-3), 70.79 (C-4), 70.56 (C-1), 62.94 (C-6), 40.03 (C-2), 34.10 (C-7). HRESIMS: Calcd for [C_15_H_18_O_7_−H]^−^: 309.0980. Found *m/z*: 309.0967.

#### 3.2.2 1,5-Anhydro-4,6-*O*-benzylidene-2-*C-*carboxymethyl-2-deoxy-d-glucitol (**15**)

A methanolic 0.03 M MaOMe (0.6 mL, 0.018 mmol) solution was added to the benzoate derivative **14**^19^ (60 mg, 0.15 mmol) in THF–MeOH (1:4 5 mL) and the reaction mixture was stirred overnight at room temperature. Afterwards, the reaction mixture was neutralised with Amberlite IR-120 (H^+^) ion-exchange resin, filtered and the filtrate concentrated under reduced pressure and co-evaporated with water (5 × 5 mL). The residue was purified by FCC (20:1:0.02 CH_2_Cl_2_–MeOH–AcOH) to give the crystalline acid **15** (33 mg, 75%): mp 183–185 °C; *R*_f_ 0.24 (20:1:0.02 CH_2_Cl_2_–MeOH–AcOH); $[\alpha ]{\;}_{\text{D}}^{25}$ −209.0 (*c* 1.0, MeOH); ^1^H NMR (CD_3_OD, 500 MHz): *δ* 7.50–7.33 (m, 5H, Ph), 5.58 (s, 1H, PhC*H*), 4.20 (dd, 1H, *J*_5,6a_ 5.0, *J*_6a,6b_ 10.3 Hz, H-6a), 4.02 (dd, 1H, *J*_1a,2_ 4.7, *J*_1a,1b_ 11.4 Hz, H-1a), 3.70 (t, 1H, *J*_6a,6b_ 10.3 Hz, H-6b), 3.50–3.45 (m, 2H, H-3, H-4), 3.37–3.28 (m, 2H, H-1b, H-5), 2.77 (dd, 1H, *J*_2,7a_ 3.0, *J*_7a,7b_ 15.8 Hz, H-7a), 2.21–2.14 (m, 1H, H-2), 2.11 (dd, 1H, H-7b); ^13^C NMR (CD_3_OD, 125 MHz): *δ* 176.06 (C=O), 139.32–127.56 (C-Ph), 103.06 (Ph*C*H), 84.55 (C-4), 73.57 (C-3), 73.17 (C-5), 71.46 (C-1), 69.84 (C-6), 41.88 (C-2), 33.58 (C-7). HRESIMS: Calcd for [C_15_H_18_O_6_−H]^−^: 293.1031. Found *m/z*: 293.1030.

#### 3.2.3 1,5-Anhydro-2-*C-*carboxymethyl-2-deoxy-d-glucitol (**6**)

A solution of the benzylidene derivative **15** (58 mg, 0.20 mmol) in AcOH (10 mL) containing 10% palladium on carbon (29 mg) was stirred under a slight overpressure of hydrogen at room temperature for 3 h. The reaction mixture was filtered through a pad of Celite and concentrated under reduced pressure. The resulting residue was purified by an RPC C18 column (10% MeOH) to furnish the carboxylic acid **6** (32 mg, 80%): *R*_f_ 0.40 (10% MeOH); $[\alpha ]{\;}_{\text{D}}^{25}$ +379.6 (*c* 0.5, MeOH); ^1^H NMR (CD_3_OD, 500 MHz): *δ* 3.99 (dd, 1H, *J*_1a,2_ 3.9, *J*_1a,1b_ 11.5 Hz, H-1a), 3.83 (dd, 1H, *J*_5,6a_ 2.1, *J*_6a,6b_ 11.8 Hz, H-6a), 3.62 (dd, 1H, H-6b), 3.23 (t, 1H, *J*_3,4_ = *J*_4,5_ = 8.6 Hz, H-4), 3.19–3.15 (m, 3H, H-1b, H-3, H-5), 2.77–2.71 (m, 1H, H-7a), 2.08–2.00 (m, 2H, H-2, H-7b); ^13^C NMR (CD_3_OD, 125 MHz): *δ* 176.44 (C=O), 82.71 (C-5), 77.59 (C-3), 73.12 (C-4), 70.81 (C-1), 63.26 (C-6), 41.34 (C-2), 33.83 (C-7). HRESIMS: Calcd for [C_8_H_14_O_6_−H]^−^: 205.0718. Found *m/z*: 205.0724.

#### 3.2.4 1,5-Anhydro-2-*C-*(carboxymethyl *N*-hydroxyamide)-2-deoxy-d-glucitol (**8**)

10% Palladium on carbon (40 mg) was added to a solution of the benzyloxyamide **17**^18^ (40 mg, 0.10 mmol) in AcOH (10 mL). The reaction mixture was stirred under a slight over pressure of hydrogen at room temperature for 4 h. After filtration through a pad of Celite the solvent was concentrated under reduced pressure. The resulting residue was purified by an RPC C18 column (10% MeOH) to furnish the hydroxamic acid **8** (13.6 mg, 62%): *R*_f_ 0.38 (10% MeOH); $[\alpha ]{\;}_{\text{D}}^{25}$ +38.9 (*c* 1.3, MeOH); ^1^H NMR (CD_3_OD, 500 MHz): *δ* 3.92 (dd, 1H, *J*_1a,2_ 4.6, *J*_1a,1b_ 11.5 Hz, H-1a), 3.83 (dd, 1H, *J*_5,6a_ 1.9, *J*_6a,6b_ 11.8 Hz, H-6a), 3.61 (dd, 1H, H-6b), 3.24–3.13 (m, 4H, H-1b, H-3, H-4, H-5), 2.54 (dd, 1H, *J*_2,7a_ 3.9, *J*_7a,7b_ 14.3 Hz, H-7a), 2.06–1.95 (m, 1H, H-2), 1.84 (dd, 1H, H-7b); ^13^C NMR (CD_3_OD, 125 MHz): *δ* 171.44 (C=O), 82.72, 77.99, 73.08, 70.66 (C-1), 63.26 (C-6), 41.64 (C-2), 32.66 (C-7). HRESIMS: Calcd for [C_8_H_15_NO_6_+Na]^+^: 244.0792. Found *m/z*: 244.0795.

#### 3.2.5 1,3,4,6-Tetra-*O*-acetyl-2-*C*-allyl-2-deoxy-α-d-glucopyranose (**19**)

A solution of the known^22^ 1,6-anhydro derivative **18** (0.865 g, 3.2 mmol) in Ac_2_O–trifluoroacetic acid (9:1, 20 mL) was stirred at room temperature overnight, whereafter it was neutralised with a solution of satd NaHCO_3_. The aqueous solution was extracted with CH_2_Cl_2_ (2 × 200 mL) and the organic extracts were combined, washed with H_2_O (200 mL), brine (200 mL), dried with MgSO_4_ and then concentrated under reduced pressure. The residue was purified by FCC (5:1→2:1 light petroleum–EtOAc) to give the tetraacetate **19** as white needles (1.17 g, 98%): mp 99–101 °C (from 10:1 light petroleum–EtOH); *R*_f_ 0.20 (1:1 light petroleum–EtOAc); $[\alpha ]{\;}_{\text{D}}^{25}$ +123.0 (*c* 1.0, CHCl_3_); ^1^H NMR (CDCl_3_, 500 MHz): *δ* 6.06 (d, 1H, *J*_1,2_ 3.1 Hz, H-1), 5.65–5.57 (m, 1H, H-8), 5.20 (t, 1H, *J*_2,3_ = *J*_3,4_ = 10.8 Hz, H-3), 5.01–4.91 (m, 3H, H-4, H-9a,b), 4.23 (dd, 1H, *J*_5,6a_ 4.0, *J*_6a,6b_ 12.4 Hz, H-6a), 3.97–3.93 (m, 2H, H-5, H-6b), 2.16–2.12 (m, 2H, H-2, H-7a), 2.09, 2.00, 1.97, 1.96 (4 × s, 12H, 4 × CH_3_CO), 1.97–1.93 (m, 1H, H-7b); ^13^C NMR (CDCl_3_, 125 MHz): *δ* 169.78, 169.60, 168.83, 167.96 (4 × C=O), 133.06 (C-8), 116.54 (C-9), 90.68 (C-1), 70.81 (C-3), 68.73 (C-5), 68.09 (C-4), 60.90 (C-6), 41.89 (C-2), 30.71 (C-7), 19.82, 19.76, 19.70, 19.53, (4 × *C*H_3_CO). HRESIMS: Calcd for [C_17_H_24_O_9_+Na]^+^: 395.1313. Found *m/z*: 395.1298.

#### 3.2.6 1,3,4,6-Tetra-*O*-acetyl-2-deoxy-2-*C*-formylmethyl-α-d-glucopyranose (**20**)

Ozone was passed through a solution of the allyl compound **19** (150 mg, 0.403 mmol) in CH_2_Cl_2_ (20 mL) at −78 °C until the solution turned blue. The excess ozone was removed by a stream of argon until the solution was clear and then followed by the addition of triphenylphosphine (264.3 mg, 1.01 mmol). The mixture was allowed to warm to room temperature for 2 h, concentrated under reduced pressure and purified by RBC (6:1→2:1 light petroleum–EtOAc) to give the aldehyde **20** (84 mg, 88%): *R*_f_ 0.28 (1:1 light petroleum–EtOAc); $[\alpha ]{\;}_{\text{D}}^{25}$ +172.7 (*c* 0.6, CHCl_3_); ^1^H NMR (CDCl_3_, 500 MHz): *δ* 9.61 (t, 1H, *J* 1.2 Hz, HC=O), 6.20 (d, 1H, *J*_1,2_ 3.4 Hz, H-1), 5.18 (dd, 1H, *J*_2,3_ 11.4, *J*_3,4_ 9.5 Hz, H-3), 5.03 (t, 1H, *J*_4,5_ 9.5 Hz, H-4), 4.23 (dd, 1H, *J*_5,6a_ 4.6, *J*_6a,6b_ 13.0 Hz, H-6a), 4.01–3.97 (m, 2H, H-5, H-6b), 2.76–2.70 (m, 1H, H-2), 2.38 (m, 2H, H-7a, H-7b), 2.09, 2.02, 19.96, 19.94 (4 × s, 12H, 4 × CH_3_CO); ^13^C NMR (CDCl_3_, 125 MHz): *δ* 197.80 (HC=O), 169.70, 169.52, 168.66, 167.88 (4 × C=O), 90.86 (C-1), 70.18 (C-3), 68.84 (C-5), 67.80 (C-4), 60.73 (C-6), 41.42 (C-7), 36.92 (C-2), 19.85, 19.80, 19.70, 19.62, (4 × *C*H_3_CO). HRESIMS: Calcd for [C_16_H_22_O_10_+Na]^+^: 397.1313. Found *m/z*: 397.1298.

#### 3.2.7 1,3,4,6-Tetra-*O*-acetyl-2-*C*-carboxymethyl-2-deoxy-α-d-glucopyranose (**21**)

A solution of sodium chlorite (2.58 g, 28.56 mmol) and sodium dihydrogen phosphate (3.92 g, 32.63 mmol) in water (20 mL) was added dropwise to a solution of the aldehyde **20** (724 mg, 1.931 mmol) in *tert*-BuOH (56.7 mL, 604 mmol) and amylene (17 mL, 203 mmol). The reaction mixture was stirred for 1 h then diluted with ice water and extracted with EtOAc (2 × 50 mL). The combined organic extracts were washed with brine (30 mL), dried (Na_2_SO_4_), and concentrated under reduced pressure. The residue was purified by FCC (1:1:0.02 light petroleum–EtOAc–AcOH) to furnish the acid **21** (709 mg, 94%): *R*_f_ 0.27 (1:1:0.02 light petroleum–EtOAc–AcOH); $[\alpha ]{\;}_{\text{D}}^{25}$ +88.3 (*c* 1.3, CHCl_3_); ^1^H NMR (CDCl_3_, 500 MHz): *δ* 6.25 (d, 1H, *J*_1,2_ 3.4 Hz, H-1), 5.22 (dd, 1H, *J*_2,3_ 11.4, *J*_3,4_ 9.5 Hz, H-3), 5.03 (t, 1H, *J*_4,5_ 9.5 Hz, H-4), 4.28 (dd, 1H, *J*_5,6a_ 4.1, *J*_6a,6b_ 12.4 Hz, H-6a), 4.08–4.02 (m, 2H, H-5, H-6b), 2.61–2.56 (m, 1H, H-2), 2.34 (dd, 1H, *J*_2,7a_ 5.8, *J*_7a,7b_ 16.3 Hz, H-7a), 2.27 (dd, 1H, H-7b), 2.04, 2.01, 2.00, 19.99 (4 × s, 12H, 4 × CH_3_CO); ^13^C NMR (CDCl_3_, 125 MHz): *δ* 174.59, 172.40, 172.14, 171.44, 170.75 (5 × C=O), 93.16 (C-1), 72.67 (C-3), 71.13 (C-5), 70.45 (C-4), 63.16 (C-6), 41.79 (C-2), 32.98 (C-7), 20.79, 20.75, 20.70, 20.65 (4 × *C*H_3_CO). HRESIMS: Calcd for [C_16_H_22_O_11_−H]^−^: 389.1089. Found *m/z*: 389.1085.

#### 3.2.8 2-*C*-Carboxymethyl-2-deoxy-d-glucopyranose (**9**)

To a solution of benzoylated compound **21** (93 mg, 0.238 mmol) in MeOH (2 mL) was added 0.03 M sodium methoxide in MeOH (6.2 mL, 0.186 mmol) at room temperature. After 48 h, the reaction mixture was neutralised with Amberlite IR-120 (H^+^) ion-exchange resin, filtered and the filtrate was concentrated under reduced pressure; followed by co-evaporation with water (5 × 5 mL). The residue was purified by FCC (3:1:0.02 CH_2_Cl_2_–MeOH–AcOH) to give the carboxylic acid **9** as an α:β (1.5:1) mixture (27 mg, 51%): *R*_f_ 0.25 (3:1:0.02 CH_2_Cl_2_–MeOH–AcOH); ^1^H NMR (CD_3_OD, 500 MHz): *δ* 5.23 (d, 1H, *J*_1,2_ 3.1 Hz, H-1α), 4.63 (d, *J*_1,2_ 8.6 Hz, H-1β), 3.85 (dd, *J*_5,6a_ 1.9, *J*_6a,6b_ 11.7 Hz, H-6aβ), 3.80–3.76 (m, 2H, H-5, H-6aα), 3.70 (dd, *J*_6a,6b_ 11.4 Hz, H-6bα), 3.66 (dd, H-6bβ), 3.51 (dd, *J*_2,3_ 10.9, *J*_3,4_ 8.9 Hz, H-3α), 3.36 (dd, *J*_2,3_ 10.7, *J*_3,4_ 8.1 Hz, H-3β), 3.32–3.21 (m, 3H, H-4α, H-4β, H-5β), 2.71 (dd, *J*_2,7a_ 3.3, *J*_7a,7b_ 16.6 Hz, H-7aα), 2.60 (dd, *J*_2,7a_ 4.1, *J*_7a,7b_ 16.1 Hz, H-7aβ), 2.46 (dd, H-7bβ), 2.39 (dd, H-7bα), 2.08–2.03 (m, 1H, H-2α), 1.91–1.85 (m, 1H, H-2β); ^13^C NMR (CD_3_OD, 125 MHz): *δ* 177.02, 175.55 (2 × C=O), 98.23 (C-1β), 93.79 (C-1α), 77.99 (C-5β), 76.06 (C-3β), 73.25 (C-5α), 73.17 (C-3α), 72.90 (C-4), 63.06 (C-6β), 63.02 (C-6α) 47.62 (C-2β), 44.83 (C-2α), 33.62 (C-7α), 33.51 (C-7β). HRESIMS: Calcd for [C_8_H_14_O_7_−H]^−^: 221.0667. Found *m/z*: 221.0659.

#### 3.2.9 Phenyl 3,4,6-tri-*O*-acetyl-2-*C*-allyl-2-deoxy-1-thio-α- and β-d-glucopyranoside (**22**) and (**23**)

To a stirred solution of the tetraacetate **19** (200 mg, 0.537 mmol) in freshly distilled CH_2_Cl_2_ (10 mL) at room temperature under argon was added thiophenol (110 μL, 1.074 mmol) and boron trifluoride diethyl etherate (270 μL, 2.148 mmol). The resulting mixture was heated to reflux for 3 h, cooled to room temperature, and then diluted with CH_2_Cl_2_ (10 mL), washed with satd NaHCO_3_ (10 mL), brine (10 mL), dried (Na_2_SO_4_), and concentrated under reduced pressure. RBC (10:1→4:1 light petroleum–EtOAc) of the residue provided the α-anomer **22** (95 mg, 48%), the β-anomer **23** (25.7 mg, 13%), as well as, an α/β mixture (51.5 mg, 26%).

α-Anomer **22**: *R*_f_ 0.28 (4:1 light petroleum–EtOAc); $[\alpha ]{\;}_{\text{D}}^{25}$ +281.0 (*c* 1.0, CHCl_3_); ^1^H NMR (CDCl_3_, 500 MHz): *δ* 7.47–7.27 (m, 5H, Ph) 5.76–5.68 (m, 1H, H-8), 5.46 (d, 1H, *J*_1,2_ 4.9 Hz, H-1), 5.23–5.18 (m, 2H, H-3, H-9a), 5.08 (dd, 1H, *J* 9.8 Hz, H-9b), 5.00 (t, 1H, *J*_3,4_ = *J*_4,5_ = 10.2 Hz, H-4), 4.63–4.60 (m, 1H, H-5), 4.31 (dd, 1H, *J*_5,6a_ 5.2, *J*_6a,6b_ 12.3 Hz, H-6a), 4.01 (dd, 1H, H-6b), 2.44–2.38 (m, 1H, H-2), 2.32–2.26 (m, 1H, H-7a), 2.25–2.16 (m, 1H, H-7b) 2.05, 2.04, 2.03 (3 × s, 12H, 3 × CH_3_CO); ^13^C NMR (CDCl_3_, 125 MHz): *δ* 170.63, 170.31, 169.97 (3 × C=O), 134.06 (C-8), 133.57–127.64 (C-Ph), 117.94 (C-9), 88.09 (C-1), 72.46 (C-3), 69.98 (C-4), 68.80 (C-5), 62.41 (C-6), 45.04 (C-2), 33.03 (C-7), 20.73, 20.72, 20.69 (3 × *C*H_3_CO). HRESIMS: Calcd for [C_21_H_26_O_7_S+Na]^+^: 445.1291. Found *m/z*: 445.1294.

β-Anomer **23**: *R*_f_ 0.24 (4:1 light petroleum–EtOAc); $[\alpha ]{\;}_{\text{D}}^{25}$ +60.0 (*c* 0.2, CHCl_3_); ^1^H NMR (CDCl_3_, 500 MHz): *δ* 7.56–7.31 (m, 5H, Ph) 5.80–5.71 (m, 1H, H-8), 5.14–5.08 (m, 3H, H-3, H-9a, H-9b), 4.94 (dd, 1H, *J*_3,4_ = *J*_4,5_ = 10.0 Hz, H-4), 4.55 (d, 1H, *J*_1,2_ 10.9 Hz, H-1), 4.24 (dd, 1H, *J*_5,6a_ 5.6, *J*_6a,6b_ 12.1 Hz, H-6a), 4.13 (dd, 1H, H-6b), 3.65–3.60 (m, 1H, H-5), 2.45–2.40 (m, 1H, H-7a), 2.34–2.29 (m, 1H, H-7b), 2.07 (s, 3H, CH_3_CO), 2.06–2.02 (m, 1H, H-2), 2.01, 2.00 (2 × s, 6H, 2 × CH_3_CO); ^13^C NMR (CDCl_3_, 125 MHz): *δ* 170.71, 170.29, 169.94 (3 × C=O), 132.47 (C-8), 132.79–128.11 (C-Ph), 118.93 (C-9), 86.45 (C-1), 75.32 (C-5), 73.16 (C-3), 69. 86 (C-4), 62.70 (C-6), 43.81 (C-2), 32.05 (C-7), 20.79, 20.75, 20.70 (3 × *C*H_3_CO).

#### 3.2.10 Phenyl 3,4,6-tri-*O*-acetyl-2-deoxy-2-*C*-formylmethyl-1-thio-α-d-glucopyranoside (**24**)

This compound was prepared from the allyl derivative **22** (95 mg, 0.225 mmol) and then quenched with triphenylphosphine (147 mg, 0.562 mmol) essentially as described for **20**. RBC (6:1→2:1 light petroleum–EtOAc) of the residue yielded the aldehyde **24** (84 mg, 88%): *R*_f_ 0.21 (2:1 light petroleum–EtOAc); $[\alpha ]{\;}_{\text{D}}^{25}$ +268.27 (*c* 0.9, CHCl_3_); ^1^H NMR (CDCl_3_, 500 MHz): *δ* 9.74 (s, 1H, HC=O), 7.44–7.28 (m, 5H, Ph), 5.75 (d, 1H, *J*_1,2_ 5.1 Hz, H-1), 5.16 (dd, 1H, *J*_2,3_ 11.5, *J*_3,4_ 9.5 Hz, H-3), 5.04 (t, 1H, *J*_4,5_ 9.5 Hz, H-4), 4.61–4.56 (m, 1H, H-5), 4.32 (dd, 1H, *J*_5,6a_ 5.1, *J*_6a,6b_ 12.3 Hz, H-6a), 4.05 (dd, 1H, H-6b), 3.03–2.95 (m, 1H, H-2), 2.76 (dd, 1H, *J*_2,7a_ 8.1, *J*_7a,7b_ 18.3 Hz, H-7a), 2.61 (dd, 1H, H-7b), 2.07, 2.04, 2.02 (3 × s, 9H, 3 × CH_3_CO); ^13^C NMR (CDCl_3_, 125 MHz): *δ* 198.86 (HC=O), 170.63, 170.31, 169.85 (3 × C=O), 132.96–127.85 (C-Ph), 87.52 (C-1), 71.92 (C-3), 69.54 (C-4), 68.62 (C-5), 62.24 (C-6), 43.37 (C-7), 39.7 3 (C-2), 20.72, 20.69, 20.67 (3 × *C*H_3_CO). HRESIMS: Calcd for [C_20_H_24_O_8_S+Na]^+^: 447.1084. Found *m/z*: 447.1096.

#### 3.2.11 Phenyl 3,4,6-tri-*O*-acetyl-2-deoxy-2-*C*-formylmethyl-1-thio-β-d-glucopyranoside (**25**)

This compound was prepared from the allyl derivative **23** (25 mg, 0.059 mmol) essentially as described for the previous α-derivative **24**. However, dimethyl sulfide (130 μL, 0.177 mmol) was used in place of triphenylphosphine. The residue was purified by RBC (6:1→2:1 light petroleum–EtOAc) to afford the aldehyde **25** (21.8 mg, 87%): *R*_f_ 0.21 (2:1 light petroleum–EtOAc); $[\alpha ]{\;}_{\text{D}}^{25}$ +11.0 (*c* 1.5, CHCl_3_); ^1^H NMR (CDCl_3_, 500 MHz): *δ* 9.59 (s, 1H, HC=O), 7.53–7.31 (m, 5H, Ph), 5.15 (dd, 1H, *J*_2,3_ 10.7, *J*_3,4_ 9.5 Hz, H-3), 4.96 (t, 1H, *J*_4,5_ 9.5 Hz, H-4), 4.84 (d, 1H, *J*_1,2_ 10.7 Hz, H-1), 4.27 (dd, 1H, *J*_5,6a_ 5.3, *J*_6a,6b_ 12.2 Hz, H-6a), 4.17 (dd, 1H, H-6b), 3.76–3.71 (m, 1H, H-5), 2.84 (dd, 1H, *J*_2,7a_ 3.8, *J*_7a,7b_ 16.4 Hz, H-7a), 2.57 (dd, 1H, H-7b), 2.45–2.38 (m, 1H, H-2), 2.10, 2.01, 1.97 (3 × s, 9H, 3 × CH_3_CO); ^13^C NMR (CDCl_3_, 125 MHz): *δ* 198.94 (HC=O), 170.69, 170.31, 169.82 (3 × C=O), 132.87–128.45 (C-Ph), 86.51 (C-1), 75.67 (C-5), 74.49 (C-3), 69.26 (C-4), 62.47 (C-6), 43.09 (C-7), 40.51 (C-2), 20.81, 20.67, 20.61 (3 × *C*H_3_CO). HRESIMS: Calcd for [C_20_H_24_O_8_S+Na]^+^: 447.1084. Found *m/z*: 447.1096.

#### 3.2.12 Phenyl 3,4,6-tri-*O*-acetyl-2-*C*-carboxymethyl-2-deoxy-1-thio-α-d-glucopyranoside (**26**)

Pinnick^21^ oxidation of the aldehyde **24** (0.383 g, 0.902 mmol) in the presence of sodium chlorite (1.20 g, 13.04 mmol), sodium dihydrogen phosphate (1.83 g, 15.24 mmol), *tert*-BuOH (26.5 mL, 282 mmol), amylene (7.96 mL, 94.71 mmol) and water (10 mL), essentially as described for compound **21,** furnished a crude residue of **26**. This residue was purified by FCC (1:1:0.02 hexane–Et_2_O–AcOH) to give the white crystalline carboxylic acid **26** (0.369 g, 93%): mp 95–98 °C; *R*_f_ 0.25 (1:1:0.02 hexane–Et_2_O–AcOH); $[\alpha ]{\;}_{\text{D}}^{25}$ +195.0 (*c* 1.0, CHCl_3_); ^1^H NMR (CDCl_3_, 500 MHz): *δ* 7.46–7.27 (m, 5H, Ph), 5.77 (d, 1H, *J*_1,2_ 5.1 Hz, H-1), 5.18 (dd, 1H, *J*_2,3_11.7, *J*_3,4_ 9.1 Hz, H-3), 5.03 (dd, 1H, *J*_4,5_ 10.1 Hz, H-4), 4.61–4.56 (m, 1H, H-5), 4.32 (dd, 1H, *J*_5,6a_ 5.1, *J*_6a,6b_ 12.3 Hz, H-6a), 4.05 (dd, 1H, H-6b), 2.90–2.85 (m, 1H, H-2), 2.61 (dd, 1H, *J*_2,7a_ 8.4, *J*_7a,7b_ 17.0 Hz, H-7a), 2.50 (dd, 1H, H-7b), 2.05, 2.04, 2.02 (3 × s, 9H, 3 × CH_3_CO); ^13^C NMR (CDCl_3_, 125 MHz): *δ* 176.64, 170.86, 170.43, 170.03 (4 × C=O), 133.10–127.79 (C-Ph), 87.65 (C-1), 71.94 (C-3), 69.75 (C-4), 68.65 (C-5), 62.30 (C-6), 41.70 (C-2), 33.78 (C-7), 20.84, 20.70, 20.61 (3 × *C*H_3_CO). HRESIMS: Calcd for [C_20_H_24_O_9_S−H]^−^: 439.1068. Found *m/z*: 439.1085.

#### 3.2.13 Phenyl 3,4,6-tri-*O*-acetyl-2-*C*-carboxymethyl-2-deoxy-1-thio-β-d-glucopyranoside (**27**)

Pinnick^21^ oxidation of the aldehyde **25** (20 mg, 0.047 mmol) in the presence of sodium chlorite (63 mg, 0.695 mmol), sodium dihydrogen phosphate (95 mg, 0.794 mmol), *tert*-BuOH (1.40 mL, 14.71 mmol), amylene (413.5 μL, 4.94 mmol) and water (10 mL), essentially as described for compound **21**, gave the crude compound **27**. This material was purified by FCC (1:1:0.02 hexane–Et_2_O–AcOH) and gave the acid **27** as white crystals (19.4 mg, 92%): mp 95–98 °C; *R*_f_ 0.25 (1:1:0.02 hexane–Et_2_O–AcOH); $[\alpha ]{\;}_{\text{D}}^{25}$ +10 (*c* 1.0, CHCl_3_); ^1^H NMR (CDCl_3_, 500 MHz): *δ* 7.50–7.31 (m, 5H, Ph), 5.26 (dd, 1H, *J*_2,3_ 10.6 Hz, *J*_3,4_ 9.3, H-3), 4.95 (t, 1H, *J*_4,5_ 9.3 Hz, H-4), 4.92 (d, 1H, *J*_1,2_ 10.9 Hz, H-1), 4.25 (dd, 1H, *J*_5,6a_ 5.3, *J*_6a,6b_ 12.2 Hz, H-6a), 4.17 (dd, 1H, H-6b), 3.75–3.71 (m, 1H, H-5), 2.69 (dd, 1H, *J*_2,7a_ 4.0, *J*_7a,7b_ 17.1 Hz, H-7a), 2.61 (dd, 1H, H-7b), 2.35–2.28 (m, 1H, H-2), 2.09, 2.01, 1.99 (3 × s, 9H, 3 × CH_3_CO); ^13^C NMR (CDCl_3_, 125 MHz): *δ* 176.26, 170.75, 170.49, 169.85 (4 × C=O), 132.96–128.37 (C-Ph), 86.24 (C-1), 75.59 (C-5), 73.97 (C-3), 69.43 (C-4), 62.53 (C-6), 41.93 (C-2), 32.94 (C-7), 20.81, 20.70, 20.61 (3 × *C*H_3_CO). HRESIMS: Calcd for [C_20_H_24_O_9_S−H]^−^: 439.1068. Found *m/z*: 439.1085.

#### 3.2.14 Phenyl 2-*C*-carboxymethyl-2-deoxy-1-thio-α-d-glucopyranoside (**10**)

To a stirred mixture of the triacetate **26** (75 mg, 0.170 mmol) in acetone (10 mL) at 56 °C was added dropwise a solution of concentrated hydrochloric acid (1 mL) in water (1.8 mL). Stirring was continued overnight at 56 °C, whereafter the mixture was neutralised with TEA, concentrated under reduced pressure and co-evaporated with toluene (2 × 5 mL). The residue was purified by an RPC C18 column (55% MeOH) to furnish the carboxylic acid as white needles **10** (40 mg, 75%): mp 144–146 °C; *R*_f_ 0.38 (55% MeOH); $[\alpha ]{\;}_{\text{D}}^{25}$ +247 (*c* 1.0, MeOH); ^1^H NMR (CD_3_OD, 500 MHz): *δ* 7.49–7.26 (m, 5H, Ph), 5.61 (d, 1H, *J*_1,2_ 4.5 Hz, H-1), 4.12–4.09 (m, 1H, H-5), 3.80 (dd, 1H, *J*_6a,6b_ 12.0 Hz, H-6a), 3.76 (dd, 1H, *J*_5,6b_ 4.9 Hz, H-6b), 3.42–3.34 (m, 2H, H-3, H-4), 2.88 (dd, 1H, *J*_2,7a_ 3.7, *J*_7a,7b_ 16.0 Hz, H-7a), 2.56–2.46 (m, 2H, H-2, H-7b); ^13^C NMR (CD_3_OD, 125 MHz): *δ* 174.48 (COOH), 136.11–128.54, (C-Ph), 90.54 (C-1), 75.13 (C-5), 74.09, 72.93, 62.56 (C-6), 45.29 (C-2), 34.85 (C-7). HRESIMS: Calcd for [C_14_H_18_O_6_S−H]^−^: 313.0751. Found *m/z*: 313.0766.

#### 3.2.15 Phenyl 2-*C*-carboxymethyl-2-deoxy-1-thio-β-d-glucopyranoside (**11**)

A methanolic 0.03 M NaOMe (0.43 mL, 0.013 mmol) solution was added to the triacetate **27** (19 mg, 0.043 mmol) in MeOH (1 mL) and the reaction mixture was stirred at room temperature overnight. Afterwards, it was neutralised with Amberlite IR-120 (H^+^) ion-exchange resin, filtered, and the filtrate was concentrated under reduced pressure. The residue was purified by an RPC C18 column (55% MeOH) to give the triol as white needles **11** (11 mg, 81%): mp 205–208 °C; *R*_f_ 0.42 (55% MeOH); $[\alpha ]{\;}_{\text{D}}^{25}$ −50.0 (*c* 0.6, MeOH); ^1^H NMR (CD_3_OD, 500 MHz): *δ* 7.54–7.27 (m, 5H, Ph), 4.85 (d, 1H, *J*_1,2_ 10.7 Hz, H-1), 3.86 (dd, 1H, *J*_6a,6b_ 11.9 Hz, H-6a), 3.68 (dd, 1H, *J*_5,6b_ 5.4 Hz, H-6b), 3.51 (t, 1H, *J*_2,3_ = *J*_3,4_ = 8.9 Hz, H-3), 3.34–3.29 (m, 1H, H-5), 3.26 (t, 1H, *J*_4,5_ 8.9 Hz, H-4), 2.73 (dd, 1H, *J*_2,7a_ 3.2, *J*_7a,7b_ 16.6 Hz, H-7a), 2.60 (dd, 1H, H-7b), 2.04–1.96 (m, 1H, H-2); ^13^C NMR (CD_3_OD, 125 MHz): *δ* 174.59 (COOH), 135.78–128.22 (C-Ph), 88.85 (C-1), 81.82 (C-5), 77.85 (C-3), 72.83 (C-4), 63.10 (C-6), 45.59 (C-2), 36.85 (C-7). HRESIMS: Calcd for [C_14_H_18_O_6_S−H]^−^: 313.0751. Found *m/z*: 313.0766.

#### 3.2.16 Phenyl 2-(*N*-aminocarbonyl)amino-2-deoxy-1-thio-β-d-glucopyranoside (**12**)

Potassium cyanate (278 mg, 3.42 mmol) was added to a suspension of the known^25^ amino-glucopyranoside **28** (607 mg, 2.24 mmol) in water (15 mL). The mixture was stirred in total darkness at room temperature for 4 days. Whereafter, the water was evaporated to dryness under reduced pressure and the residue was co-evaporated with toluene (3 × 20 mL). RPC (25% CH_3_CN) of the residue yielded the ureido compound **12** (458 mg, 65%): mp 220–222 °C (MeOH); *R*_f_ 0.40 (25% CH_3_CN); $[\alpha ]{\;}_{\text{D}}^{25}$ −32.0 (*c* 1.5, DMSO); ^1^H NMR (DMSO, 500 MHz): *δ* 7.41–7.17 (m, 5H, Ph), 6.02 (d, 1H, *J* 8.7 Hz, NH), 5.52 (s, 2H, NH_2_), 5.07 (d, 2H, *J* 5.2 Hz, OH-3 and OH-4), 4.81 (d, 1H, *J*_1,2_ 10.3 Hz, H-1), 4.62 (dd, 1H, *J* 5.8, *J* 11.5 Hz, 6-OH), 3.70 (ddd, 1H, *J*_5,6a_ 5.3, *J*_6a,6b_ 11.8 Hz, H-6a), 3.45 (d, 1H, H-6b), 3.40 (ddd, 1H, *J*_2,3_ 9.0 Hz, H-2), 3.30 (dt, 1H, *J*_3,4_ 9.0.Hz, H-3), 3.24–3.21 (m, 1H, H-5), 3.13 (dt, 1H, *J*_4,5_ 9.0 Hz, H-4); ^13^C NMR (DMSO, 125 MHz): *δ* 158.47 (C=O), 136.17–125.85 (C-Ph), 86.56 (C-1), 80.87 (C-5), 76.04 (C-3), 70.54 (C-4), 60.97 (C-6), 54.94 (C-2). HRESIMS: Calcd for [C_13_H_18_N_2_O_5_S+H]^+^: 315.1009. Found *m/z*: 315.1012.

## Supplementary data

Supplementary data 1 Spectral data.

## Footnotes

† Compound **8** is in the literature, Jackman, J. E. et al. *J. Biol. Chem.***2000**, *275*, 11002–11009, however, no preparative details are given therein.

## References

[1] Ferguson M.A.J. (1999). J. Cell Sci..

[2] Ferguson M.A.J., Kinoshita T., Hart G.W., Varki A., Cummings R.D., Esko J.D., Freeze H.H., Stanley P., Bertozzi C.R., Hart G.W., Etzler M.E. (2008). Essential Glycobiology.

[3] Orlean P., Menon A.K. (2007). J. Lipid Res..

[4] Kinoshita T., Fujita M., Maeda Y. (2008). J. Biochem..

[5] Nagamune K., Nozaki T., Maeda Y., Ohishi K., Fukuma T., Hara T., Schwarz R.T., Sutterlin C., Brun R., Reizman H., Kinoshita T. (2000). Proc. Natl. Acad. Sci. U.S.A..

[6] Chang T., Milne K.G., Guther M.L.S., Smith T.K., Ferguson M.A.J. (2002). J. Biol. Chem..

[7] Lillico S., Field M.C., Blundell P., Coombs G.H., Mottram J.C. (2003). Mol. Biol. Cell.

[8] Hong Y., Nagamune K., Ohishi K., Morita Y.S., Ashida H., Maeda Y., Kinoshita T. (2006). FEBS Lett..

[9] Smith T.K., Crossman A., Brimacombe J.S., Ferguson M.A.J. (2004). EMBO J..

[10] Doering T.L., Masterson W.J., Englund P.T., Hart G.W. (1989). J. Biol. Chem..

[11] Sharma D.K., Smith T.K., Crossman A., Brimacombe J.S., Ferguson M.A.J. (1997). Biochem. J..

[12] Guther M.L.S., Ferguson M.A.J. (1995). EMBO J..

[13] Murakami Y., Siripanyapinoyo U., Hong Y., Kang J.Y., Ishihara S., Nakakuma H., Maeda Y., Kinoshita T. (2003). Mol. Biol. Cell.

[14] Urbaniak M.D., Crossman A., Chang T., Smith T.K., Aalten D.M.F.V., Ferguson M.A.J. (2005). J. Biol. Chem..

[15] Jacobsen F.E., Lewis J.A., Cohen S.M. (2007). ChemMedChem.

[16] Parrish D.A., Zou Z., Allen C.L., Day C.S., King S.B. (2005). Tetrahedron Lett..

[17] Crossman A., Urbaniak M.D., Ferguson M.A.J. (2008). Carbohydr. Res..

[18] Li X., Uchiyama T., Raetz C.R.H., Hindsgaul O. (2003). Org. Lett..

[19] Li X., McClerren A.L.M., Raetz C.R.H., Hindsgaul O.J. (2005). Carbohydr. Chem..

[20] Smith T.K., Crossman A., Borrissow C.N., Paterson M.J., Dix A., Brimacombe J.S., Ferguson M.A.J. (2001). EMBO J..

[21] Bal B.S., Childers W.E., Pinnick H.W. (1981). Tetrahedron.

[22] Leteux C., Veyrières A., Robert F. (1993). Carbohydr. Res..

[23] Ferrier R.J., Furneaux R.H. (1976). Carbohydr. Res..

[24] Khiar N., Fernandez I., Araujo C.S., Rodriguez J.A., Suarez B., Alvarez E. (2003). J. Org. Chem..

[25] Benakli K., Zha C., Kerns R.J. (2001). J. Am. Chem. Soc..

[26] Kovács J., Pintér I., Messmer A., Tóth G., Lendering U., Köll P. (1990). Carbohydr. Res..

[27] Baker B.R., Hullar T.L. (1965). J. Org. Chem..

