# Supplementary material for: Synthesis of potential metal-binding group compounds to examine the zinc dependency of the GPI de-N-acetylase metalloenzyme in Trypanosoma brucei
Source: Carbohydr Res. 2011 May 1;346(6):708–14. doi: 10.1016/j.carres.2011.02.004 (PMC3125106; doi:10.1016/j.carres.2011.02.004)
Supplement: Supplementary data 1 — Spectral data. [file mmc1.doc]

Supplementary Data

**Synthesis of potential metal-binding group compounds to examine the zinc dependency of the GPI de-*N*-acetylase metalloenzyme in *Trypanosoma brucei***

Nuha Z. Abdelwahab, Michael D. Urbaniak, Michael A. J. Ferguson*, Arthur T. Crossman

*Division of Biological Chemistry and Drug Discovery, College of Life Sciences, The University of Dundee, DD1 5EH Dundee, Scotland, United Kingdom*

*Corresponding author. Tel.: +44-1382-384219; e-mail: m.a.j.ferguson@dundee.ac.uk

**2-*C*-Allyl-1,5-anhydro-3-*O*-benzoyl-4,6-*O*-benzylidene-2-deoxy-D-glucitol (S1)**

The known compound **13** was processed as previously described.^18^ However, a different FCC solvent system (5:1 hexane − Et_2_O) was used to furnish the benzoylated derivative **S1**, 95% yield, as white needles: mp 76 − 79 °C; R_f_ 0.20 (5:1 hexane − Et_2_O); $[\text{α}]_{D}^{25}$-40.5 (*c* 1.0, CHCl_3_); ^1^H NMR (CDCl_3_, 500 MHz): *δ* 8.16 − 7.38 (m, 10H, 2 × Ph), 5.77 − 5.69 (m, 1H, H-8), 5.52 (s, 1H, PhC*H*), 5.36 (t, 1H, *J*_2,3_ = *J*_3,4_ = 10.3 Hz, H-3), 5.04 − 4.98 (m, 2H, H-9a, H-9b), 4.34 (dd, 1H, *J*_5,6a_ 5.0, *J*_6a,6b_ 10.4 Hz, H-6a), 4.05 (dd, 1H, *J*_1a,2_ 5.0, *J*_1a,1b_ 11.9 Hz, H-1a), 3.75 (m, 2H, H-4, H-6b ), 3.52 (ddd, 1H, *J*_4,5_ 9.4 Hz, H-5), 3.39 (t, 1H, *J*_1a,1b_ 11.9 Hz, H-1b), 2.33 − 2.27 (m, 1H, H-7a), 2.26 − 2.18 (m, 1H, H-2), 2.04 − 1.97(m, 1H, H-7b); ^13^C NMR (CDCl_3_, 125 MHz): *δ* 166.09 (Ph*C*O), 134.51 (C-8), 133.82 − 126.06 (C-Ph), 117.47 (C-9), 101.37 (Ph*C*H), 80.86 (C-4), 74.16 (C-3), 71.99 (C-5), 70.67 (C-1), 68.93 (C-6), 41.32 (C-2), 32.50 (C-7). HRESIMS: Calcd for [C_23_H_24_O_5_ + Na]^+^: 403.1697. Found *m/z*: 403.1693.

**1,5-Anhydro-3-*O*-benzoyl-4,6-*O*-benzylidene-2-deoxy-2-*C-*formylmethyl-D-glucitol (S2)**

Ozonolysis of the alkene **S1** to the aldehyde **S2**, 92% yield, was conducted as described:^19^ mp 168 − 170 °C; R_f_ 0.23 (2:1 hexane − EtOAc); $[\text{α}]_{D}^{25}$-73.5 (*c* 1.0, CHCl_3_); ^1^H NMR (CDCl_3_, 500 MHz): *δ* 9.71 (s, 1H, HC=O), 8.06 − 7.28 (m, 10H, 2 × Ph), 5.54 (s, 1H, PhC*H*), 5.35 (t, 1H, *J*_2,3_ = *J*_3,4_ = 10.5 Hz, H-3), 4.35 (dd, 1H, *J*_5,6a_ 4.9, *J*_6a,6b_ 10.5 Hz, H-6a), 4.12 (dd, 1H, *J*_1a,2_ 5.0, *J*_1a,1b_ 11.6 Hz, H-1a), 3.82 – 3.74 (m, 2H, H-4, H-6b), 3.50 − 3.51 (ddd, 1H, *J*_4,5_ 9.4 Hz, H-5), 3.39 (t, 1H, *J*_1a,1b_ 11.6 Hz, H-1b), 2.80 − 2.72 (m, 1H, H-2), 2.67 (dd, 1H, *J*_2,7a_ 4.3, *J*_7a,7b_ 18.3 Hz, H-7a), 2.38 (dd, 1H, H-7b); ^13^C NMR (CDCl_3_, 125 MHz): *δ* 199.30 (HC=O), 166.14 (Ph*C*=O), 137.05 − 125.99 (C-Ph), 101.34 (Ph*C*H), 80.45 (C-4), 73.73 (C-3), 71.99 (C-5), 70.11 (C-1), 68.76 (C-6), 41.15 (C-7), 36.52 (C-2). HRESIMS: Calcd for [C_22_H_22_O_6_ + Na]^+^: 405.1309. Found *m/z*: 405.1328.

**1,5-Anhydro-3-*O*-benzoyl-4,6-*O*-benzylidene-2-*C-*carboxymethyl-2-deoxy-D-glucitol (14)**

The aldehyde **S2** was oxidized to the carboxylic acid **14**,^19^ 95% yield, which was purified by FCC (3:1:0.02 hexane – EtOAc – AcOH) to give the crystalline acid **14**: mp 215 – 217 °C; R_f_ 0.27 (3:1:0.02 hexane – EtOAc – AcOH); $[\text{α}]_{D}^{25}$-68.0 (*c* 1.0, THF); ^1^H NMR (CDCl_3_, 500 MHz): *δ* 8.06 – 7.38 (m, 10H, 2 × Ph), 5.52 (s, 1H, PhC*H*), 5.36 (t, 1H, *J*_2,3_ = *J*_3,4_ = 10.5 Hz, H-3), 4.34 (dd, 1H, *J*_5,6a_ 4.9, *J*_6a,6b_ 10.5 Hz, H-6a), 4.13 (dd, 1H, *J*_1a,2_ 4.9, *J*_1a,1b_ 11.6 Hz, H-1a), 3.80 – 3.75 (m, 2H, H-4, H-6b), 3.54 (ddd, 1H, *J*_4,5_ 9.6 Hz, H-5), 3.44 (t, 1H, *J*_1a,1b_ 11.6 Hz, H-1b), 2.65 − 2.56 (m, 1H, H-2), 2.47 (dd, 1H, *J*_2,7a_ 4.6, *J*_7a,7b_ 16.5 Hz, H-7a), 2.21 (dd, 1H, H-7b); ^13^C NMR (CDCl_3_, 125 MHz): *δ* 176.56 (C=O), 166.06 (Ph*C*O), 137.02 – 128.12 (C-Ph), 101.34 ( Ph*C*H ), 80.48 (C-4), 73.64 (C-3), 71.96 (C-5), 70.14 (C-1), 68.75 (C-6), 38.62 (C-2), 32.61 (C-7). HRESIMS: Calcd for [C_22_H_22_O_7_ + Na]^+^: 421.1258. Found *m/z*: 421.1274.

**1,5-Anhydro-3-*O*-benzoyl-4,6-*O*-benzylidene-2-*C-*(carboxymethyl *N*-benzyloxyamide)- 2-deoxy-D-glucitol (16)**

The benzyloxyamide **16** was synthesized from the carboxylic acid **14** essentially as described,^19^ except EDAC was used as the coupling agent. The crude residue of **16** was purified by RBC (5:1→3:1→1:1 light petroleum − EtOAc) to give the crystalline benzyloxyamide **16**, 78% yield: mp 181 − 183 °C; R_f_ 0.29 (1:1 light petroleum − EtOAc); $[\text{α}]_{D}^{25}$-34.4 (*c* 1.0, CHCl_3_); ^1^H NMR (CDCl_3_, 500 MHz): *δ* 8.62 (s, 1H, NH), 7.98 − 7.25 (m, 15H, 3 × Ph), 5.44 (s, 1H, PhC*H*), 5.14 (t, 1H, *J*_2,3_ = *J*_3,4_ = 9.7 Hz, H-3), 4.71 (ABq, 2H, *J* 11.3 Hz, C*H*_2_Ph), 4.24 (dd, 1H, *J*_5,6a_ 4.9, *J*_6a,6b_ 10.5 Hz, H-6a), 4.02 (dd, 1H, *J*_1a,2_ 4.7, *J*_1a,1b_ 11.6 Hz, H-1a), 3.69 (m, 2H, H-4, H-6b), 3.39 − 3.33 (m, 1H, H-5), 3.27 (t, 1H, *J*_1a,1b_ 11.6 Hz, H-1b), 2.61 − 2.54 (m, 1H, H-2), 2.14 (dd, 1H, *J*_2,7a_ 3.7, *J*_7a,7b_ 14.6 Hz, H-7a), 1.86 (dd, 1H, H-7b); ^13^C NMR (CDCl_3_, 125 MHz): *δ* 173.51 (C=O), 166.65 (Ph*C*O), 136.10 − 125.01 (C-Ph), 100.30 ( Ph*C*H), 79.30 (C-4), 77.05 ( Ph*C*H_2_), 73.28 (C-3), 70.78 (C-5), 68.91 (C-1), 67.75 (C-6), 37.49 (C-2), 30.77 (C-7). HRESIMS: Calcd for [C_29_H_29_NO_7_ + H]^+^: 504.2017. Found *m/z*: 504.2027.

**1,5-Anhydro-3-*O*-benzoyl-2-*C-*(carboxymethyl *N*-hydroxyamide)-2-deoxy-D-glucitol (7)**

The hydrogenation of **16** to give **7** was conducted as described previously.^19^ However, the residue of **7** was purified by FCC (5:1 CHCl_3_ − MeOH) to furnish the hydroxyamide **7**, 67% yield: R_f_ 0.25 (5:1 CHCl_3_ − MeOH); $[\text{α}]_{D}^{25}$+8.1 (*c* 1.2, MeOH); ^13^C NMR (CD_3_OD, 125 MHz): *δ* 170.33, 168.24 (2 × C=O), 134.33 − 129.54 (C-Ph), 82.81 (C-5), 79.89 (C-3), 70.77 (C-4), 70.33 (C-1), 62.94 (C-6), 40.06 (C-2), 32.60 (C-7). HRESIMS: Calcd for [C_15_H_19_NO_7_ + Na]^+^: 348.1054. Found *m/z*: 348.1041.

**1,5-Anhydro-4,6-*O*-benzylidene-2-*C-*(carboxymethyl *N*-benzyloxyamide)-2-deoxy- D-glucitol (17)**

To a stirred solution of the known^19^ benzoate compound **16** (100 mg, 0.20 mmol) in THF − MeOH (1:4 5 mL) was added 0.03M NaOMe in methanol (5.2 mL, 0.156 mmol). After 48 h, the reaction mixture was neutralized with Amberlite IR-120 (H^+^) ion-exchange resin, filtered and the filtrate was concentrated under reduced pressure. The residue was partitioned between CH_2_Cl_2_ (20 mL) and water (20 mL); the aqueous phase was separated and further extracted with CH_2_Cl_2_ (2 × 20 mL). The combined CH_2_Cl_2_ extracts were washed with brine (20 mL), dried (Na_2_SO_4_), and concentrated under reduced pressure. The residue was purified by FCC (20:1 CH_2_Cl_2_ − MeOH) to give the benzyloxyamide **17**^18^ as a white amorphous powder (58 mg, 73%): R_f_ 0.25 (20:1 CH_2_Cl_2_ − MeOH); mp 190 − 192 °C; $[\text{α}]_{D}^{25}$-9.6 (*c* 1.0, 1:1 CHCl_3_ − MeOH. HRESIMS: Calcd for [C_22_H_25_NO_6_ + H]^+^ 400.1755. Found *m/z*: 400.1754.
